# RNaseH2 inhibition potentiates temozolomide response in patient derived glioblastoma cells

**DOI:** 10.1038/s41598-025-25298-5

**Published:** 2025-11-24

**Authors:** Miroslava Kissova, Judit Martinez Segarra, Tobias Solli Iveland, Marthe Vestvik, Erlend Ravlo, Wei Wang, Lars Hagen, Nina-Beate Liabakk, Maria Camara-Quilez, Miquel Arano Barenys, Ole Solheim, Bård Helge Hoff, Eirik Sundby, Magnar Bjørås, Geir Slupphaug, Torkild Visnes, Alessandro Brambilla

**Affiliations:** 1 Department of Clinical and Molecular Medicine, Norwegian University of Science and Technology NTNU, Trondheim, 7491 Norway; 2 Clinic of Laboratory Medicine, St. Olavs Hospital, Trondheim, 7491 Norway; 3 Department of Biotechnology and Nanomedicine, SINTEF Industry, Trondheim, Norway; 4 Department of Microbiology, Oslo University Hospital and University of Oslo, Oslo, Norway; 5 Department of Neuromedicine and Movement Science, NTNU, Trondheim, Norway; 6 Department of Neurosurgery, St. Olavs University Hospital, Trondheim, Norway; 7 PROMEC Core Facility for Proteomics and Modomics, Norwegian University of Science and Technology, NTNU, and the Central Norway Regional Health Authority Norway, Trondheim, 7491 Norway; 8 Department of Chemistry, Faculty of Natural Sciences, Norwegian University of Science and Technology NTNU, Trondheim, Norway; 9 Department of Material Science, Norwegian University of Science and Technology, NTNU, Trondheim, 7491 Norway; 10 Center of Embryology (CRESCO), University of Oslo, Oslo, 0313 Norway; 11 Department of Oncology, Akershus University Hospital, Lørenskog, 1478 Norway

**Keywords:** GBM, High-throughput screening (HTS), Drug screening, Cancer, Drug discovery, Molecular biology

## Abstract

Glioblastoma (GBM) is the most common and lethal form of primary brain tumor, characterized by poor prognosis and limited treatment options, with a median survival of only 12 to 15 months post-diagnosis. Despite standard therapeutic protocols involving temozolomide (TMZ) and radiotherapy, effective treatment is challenging, underscoring the need to explore alternative therapeutic strategies. One promising approach is the inhibition of DNA damage response (DDR) pathways. RNaseH2, a key enzyme involved in ribonucleotide excision repair (RER), plays a critical role in maintaining genomic stability by removing ribonucleotides misincorporated into DNA. Thus, inhibition of RNaseH2 may enhance tumor mutational burden (TMB) and potentially transform immunogenically “cold” tumors into immunogenically “hot” tumors. In this study, a high-throughput screening (HTS) assay resulted in the identification of 52 potential RNaseH2 inhibitors from a library of 71227 compounds. We selected six of these inhibitors for further investigation, evaluating their effects both alone and in combination with TMZ in commercially available U87 MG wild-type and IDH1 mutant (IDH1 WT and MUT) glioma cells and patient-derived cells established from glioma organoids (GBO-PDC). This study highlights the therapeutic potential of RNaseH2 inhibition in combination with TMZ for GBM therapy, validated in patient-derived model, offering a promising avenue for treating this highly aggressive and yet uncurable cancer.

**Supplementary Information:**

The online version contains supplementary material available at 10.1038/s41598-025-25298-5.

## Introduction

GBM is the most frequently occurring primary brain tumor and is associated with a very poor prognosis. Regardless of the current treatment, the average survival time following diagnosis is only 12 to 15 months^1,2^. The global incidence of GBM is approximately 0.59 to 5 per 100000 people, with a higher incidence rate observed in individuals over 75 years old^3,4^. A defining molecular characteristic in glioma subclassification is the driver mutation of isocitrate dehydrogenase 1 and 2 (IDH1/2)^5–7^. These enzymes catalyze the oxidative decarboxylation of isocitrate and play an essential role in the Krebs cycle and cellular homeostasis^8^. These mutants have enhanced affinity for α-ketoglutarate (2OG) over isocitrate, and thereby irreversibly convert 2OG to the D2-enantiomer of hydroxyglutarate (2OHG)^9^. 2OHG is classified as an oncometabolite^10^ and inhibits a large number of 2OG-dependent enzymes, including DNA, RNA, and histone demethylases. In gliomas, the IDH1 R132H mutant is by far the most common, accounting for approximately 90% of all IDH mutations^6^.

Current GBM therapy involves surgical resection followed by temozolomide (TMZ) injections at a dose of 75 mg/m² per day for 6 weeks, combined with 60 Gy of radiotherapy administered in 30 fractions. This is followed by five maintenance cycles of TMZ, given at 150–200 mg/m² per day for 5 days within each 28-day cycle^11^. While effective to some extent, this treatment does not cure the disease, highlighting the urgent need for alternative therapeutic approaches. A promising strategy under investigation involves targeting the DNA damage response (DDR) induced by DNA-damaging agents^12–14^. Several studies emphasize the potential of inhibiting RNaseH2 as a treatment strategy for a wide range of tumors^11,15,16^. This enzyme plays a key role in ribonucleotide excision repair (RER) and is the primary source of RNase H activity in the nucleus. It consists of one catalytic subunit (RNASEH2A) and two accessory subunits (RNASEH2B and RNASEH2C) that are essential for enzymatic activity^17,18^. Upon recognizing a ribonucleotide monophosphate (rNMP) in the context of DNA, it cuts the backbone 5´- to the ribonucleotide, thereby generating a single-strand break. DNA polymerase δ (POLD) or ε (POLE) then initiates strand displacement DNA synthesis, generating a flap structure containing the misincorporated ribonucleotide. The flap is finally cleaved by one of the endonucleases FEN1 or EXO1. DNA ligase seals the gap, thereby completing RER^19^. RNaseH2 activity is essential in eukaryotic cells and its absence leads to embryonic lethality in mice^20^. In humans, mutations in the subunits of this enzyme result in the severe phenotype of Aicardi-Goutières syndrome^21,22^.

In this study, we have developed a fluorescence-based high-throughput screening (HTS) assay to identify RNaseH2 inhibitors from a library of 71227 compounds, including candidates with prior pharmacological characterization as a part of drug repurposing strategy. Among these, we identified 52 potential enzyme inhibitors and selected six for further evaluation, both alone and in combination with TMZ, in glioma cell lines (U87 MG IDH1 WT and mutant) and in GBO-PDC. Our results indicate that combining RNaseH2 inhibitors with TMZ can enhance cytotoxic effects in glioma cell lines and in patient-derived models, suggesting that RNaseH2 inhibition is a promising approach for enhancing the effectiveness of current GBM therapies.

## Results

### Identification of RNaseH2 inhibitors

**Fig. 1:**
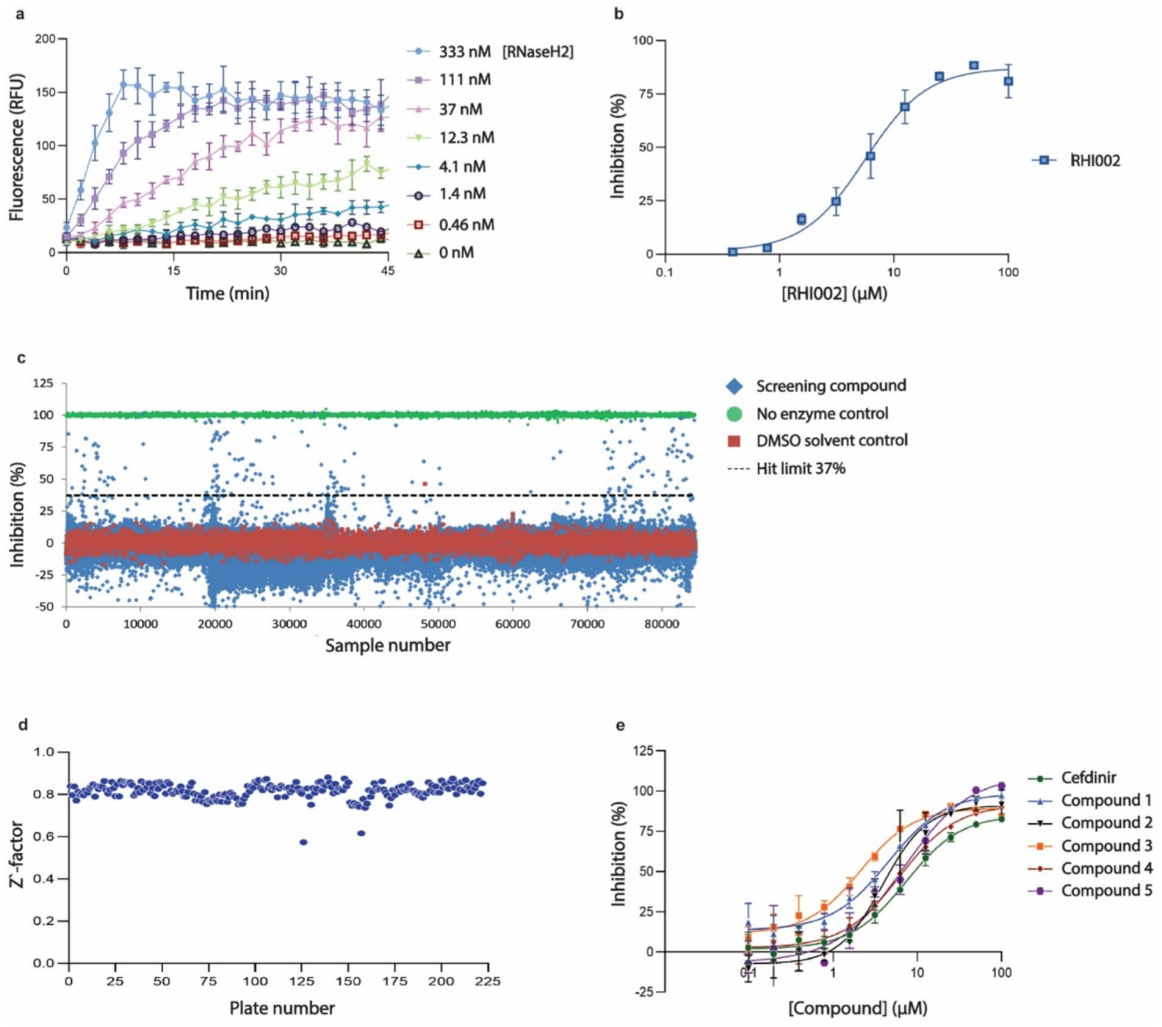
Identification of RNaseH2 inhibitors. (**a**) RNaseH2 activity assay. An 80 nM RNA/DNA hybrid substrate was incubated with the indicated concentrations of RNaseH2, and fluorescence was recorded over 45 minutes. Each data point represents the average of four technical replicates. (**b**) RNaseH2 inhibition. The indicated concentrations in RHI002 were incubated with RNaseH2 and the RNA/DNA hybrid substrate for 25 minutes. Each data point denotes the average (± SD) of two technical replicates and was fitted to a four-parameter logistic curve. (**c**) High-throughput screening for RNaseH2 inhibitors. Each symbol represents one technical replicate of 71394 screening compounds, 9495 DMSO solvent controls, and 3552 no-enzyme controls. A total of 177 compounds were identified as hits, using a hit limit defined as three standard deviations above the average of all screening compounds. (**d**) Z’-factor of high-throughput screening assay. A Z’-factor was calculated for all screening plates, based on *n* = 16 DMSO solvent controls and *n* = 16 no-enzyme controls, representing 100% and 0% inhibition, respectively. (**e**) Hit validation experiments on six candidate RNaseH2 inhibitors. RNaseH2 activity was assayed at the indicated compound concentrations. Data are presented as average ± SD of two technical replicates and were fitted to a four-parameter logistic curve.

**Fig. 2:**
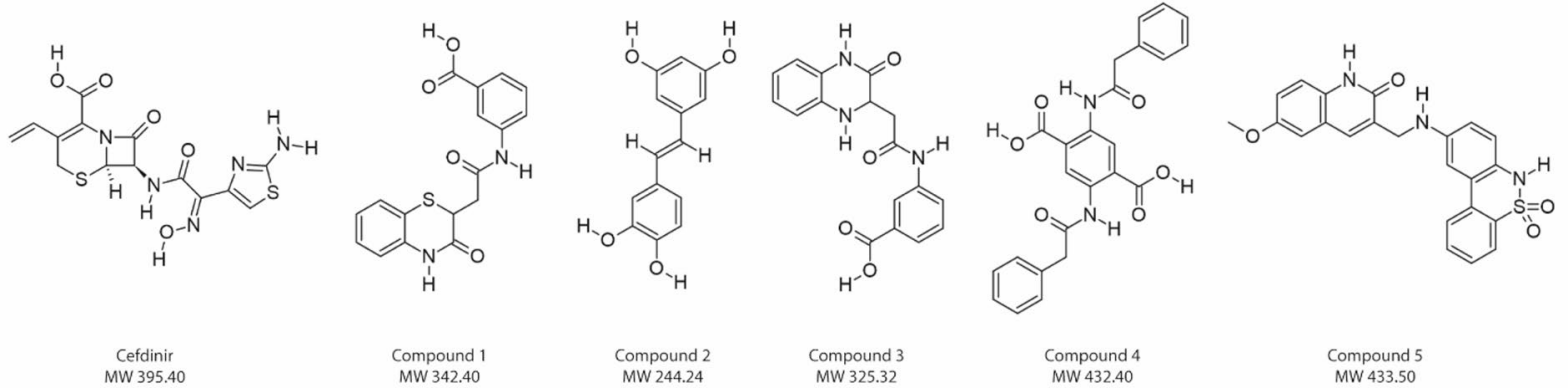
Chemical structures of RNaseH2 inhibitor candidates. Cefdinir: ((6R,7R)−7-{2-(2-Amino-thiazol-4-yl)−2-[(Z)-hydroxyimino]-acetylamino}−8-oxo-3-vinyl-5-thia-1-aza-bicyclo[4.2.0]oct-2-ene-2-carboxylic acid), Compound 1: (3-[2-(3-oxo-3,4-dihydro-2 H-1,4-benzothiazin-2-yl)acetamido]benzoic acid), Compound 2: (5-[(1E)−2-(3,4-dihydroxyphenyl)ethenyl]benzene-1,3-diol), Compound 3: (3-[2-(3-oxo-1,2,3,4-tetrahydroquinoxalin-2-yl)acetamido]benzoic acid), Compound 4: (2,5-bis(2-phenylacetamido)benzene-1,4-dicarboxylic acid), and Compound 5: (3-[[(5,5-dioxo-6 H-benzo[c][1,2]benzothiazin-9-yl)amino]methyl]−6-methoxy-1 H-quinolin-2-one).

We adapted a previously published fluorogenic assay for DNA glycosylase inhibitors^23,24^. Briefly, a biochemical activity assay was established employing a duplex RNA/DNA oligonucleotide containing an internally quenched fluorophore and a single ribonucleotide positioned so that RNaseH2-mediated incision would lead to dissociation and de-quenching. After optimizing buffer conditions and substrate concentrations (Supplementary information figure S1), we titrated RNaseH2 enzyme and observed a concentration-dependent increase in fluorescence after incubation, with a signal-to-noise ratio of ~ 20 for full substrate conversion (Fig. 1a). To assess the assay’s ability to identify inhibitors, we employed different concentrations of the known RNaseH2 inhibitor RHI002^25^ and observed a concentration-dependent decrease in fluorescence with a half-maximal inhibitory concentration (IC50) of 5.6 µM, which is close to the reported value of 16 µM (Fig. 1b). To identify inhibitors, we screened several compound libraries containing a total of 71394 chemical compounds (Fig. 1c). An average Z’-factor of 0.82 ± 0.037 (*n* = 222 plates, Fig. 1d) was achieved, suggesting that the assay was sufficiently robust to identify RNaseH2-inhibiting compounds. A total of 178 compounds were observed to reduce fluorescence by more than three standard deviations (i.e., > 37% inhibition). However, of these, 101 compounds were excluded due to frequent-hitter behavior in previous assays, and another 25 were excluded due to chemotypes associated with pan-assay interference^26^. The remaining 52 compounds were then re-tested in 11 different concentrations, of which 16 compounds were found to inhibit RNaseH2 with IC50 < 20 µM in hit validation experiments. Six of these (Fig. 2) were purchased after re-synthesis, tested, and found to inhibit RNaseH2 with IC50-values ranging from 2.1 to 7.2 µM (Fig. 1e).

### Novel candidate RNaseH2 inhibitors synergistically potentiate TMZ cytotoxicity in U87MG cells

**Fig. 3:**
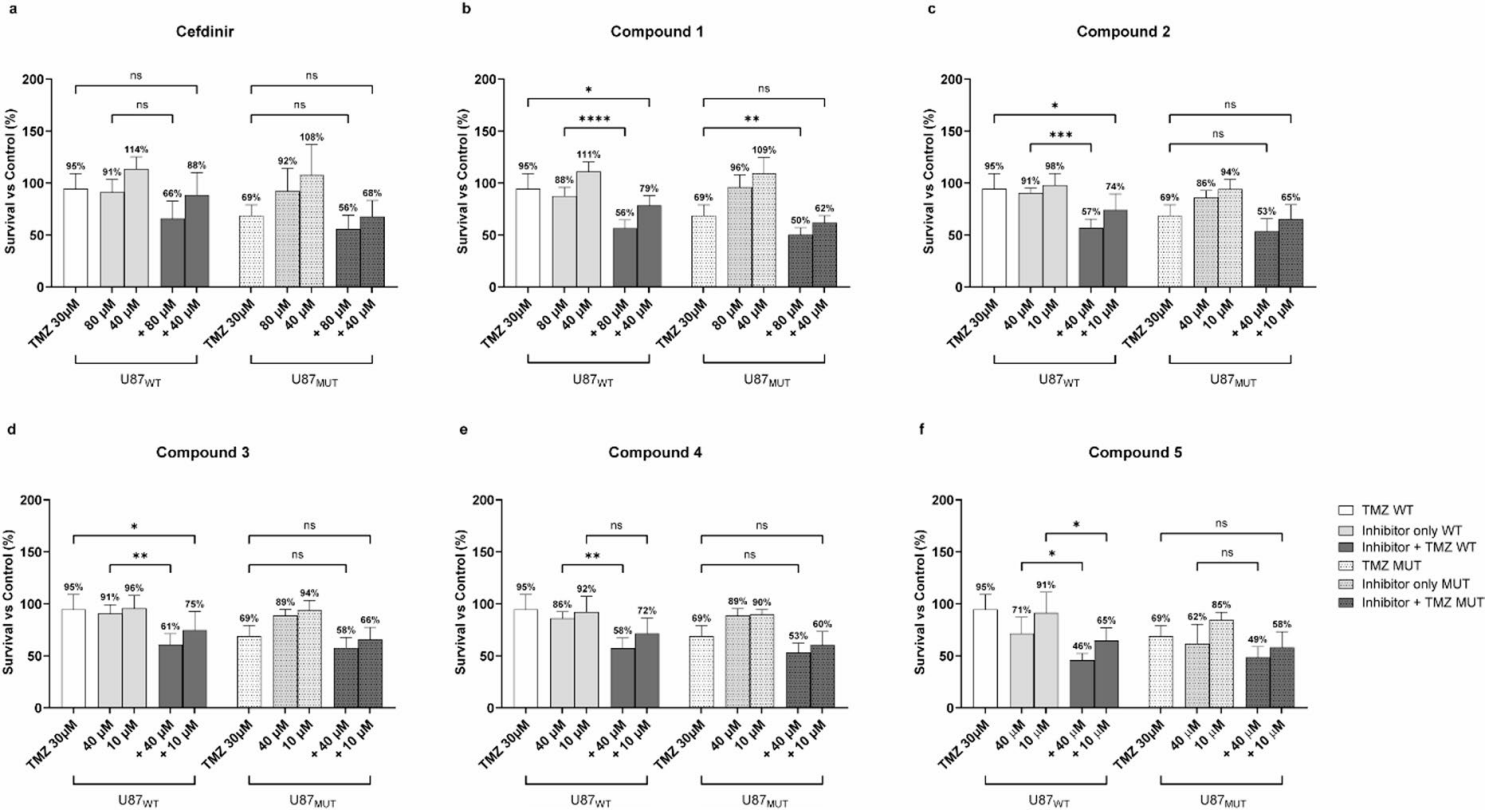
Single- and combinatorial treatments with TMZ and RNaseH2 inhibitor candidates in U87WT and U87MUT cells. Cells were treated for 72 h with two inhibitor concentrations and/or 30 µM TMZ. Data are normalized to vehicle-treated control (DMSO), and mean survival percentages are given above the bars. Error bars indicate ± SD of at least three independent experiments. Statistical significance was calculated using one-way ANOVA followed by Tukey’s post hoc test (ns = non-significant; *p = 0.01–0.05; **p = 0.001–0.01; ***p = 0.0001–0.001; ****p < 0.0001).

Following the identification of potential RNaseH2 inhibitors via high-throughput screening, we selected the most potent inhibitors for further analysis, both as single agents and in combination with TMZ (30 µM) in the glioma cell line U87MG (U87WT in the following) and U87MG cells with monoallelic expression of the IDH1 R132H mutant (U87MUT in the following). After performing a pilot experiment, the following inhibitors and concentrations were used: cefdinir and compound 1 at 80 and 40 µM; and the rest of the inhibitors were used at 40 and 10 µM (compound 2–5) (Fig. 3a-f). Compound 1, compound 2, compound 3, compound 4, and compound 5 alone did not significantly affect cell viability, but the combinatorial treatments significantly reduced viability of both U87WT and U87MUT compared to TMZ alone with at least one of the inhibitor concentrations used (Fig. 3b-f), suggesting potential cooperative cytotoxic mechanisms. In contrast, Cefdinir did not affect the viability of either U87WT or U8MUT cells when used alone or in combination with TMZ (Fig. 3a). Four of the inhibitors, compound 2, compound 3, compound 4, and compound 5, enhanced the cytotoxicity of TMZ in U87WT, but this effect was not seen in U87MUT (Fig. 3c, f). There was an overall trend that the inhibitors had a less pronounced cytotoxic effect with TMZ in the IDH1 mutant than in the wild-type cells. Potentially contributing to this could be that IDH mutant glioma cells are more sensitive to TMZ treatment alone, as supported by our findings and others^27^. To determine the potential synergy between RNaseH2 inhibitors and TMZ in glioma cytotoxicity from the initial screening data, synergy scores were calculated using SynergyFinder + software with ZIP model (Table 1). In U87WT, all combinations + TMZ were reported synergistic, whereas in U87MUT only cotreatment with compound 1 was reported synergistic. To ensure that the viability observed in the Presto Blue assay reflected the cell death, we performed a cell counting assay using Trypan Blue. While this confirmed a trend toward reduced viable cell numbers, particularly in the combination treatment (Supplementary information figure S2), the effects were generally less pronounced than those seen in the metabolic assay.

**Table 1:** Synergy scoring between RNaseH2 inhibitors and TMZ in glioma U87WT and U87MUT cell lines.

RNaseH2 Inhibitors	U87 MG IDH1 WT		U87 IDH1 MUT	
	Chemotherapy	Synergy score	Chemotherapy	Synergy score
Cefdinir	+ TMZ 30 µM	15.61	+ TMZ 30 µM	5.64
Compound 1		23.07		13.25
Compound 2		18.18		−4.36
Compound 3		21.04		1.14
Compound 4		19.10		5.43
Compound 5		20.62		−3.16

Synergistic combinations are marked in green (consensus synergistic value > 10; additive interaction − 10 to + 10; antagonism < −10).

**Fig. 4:**
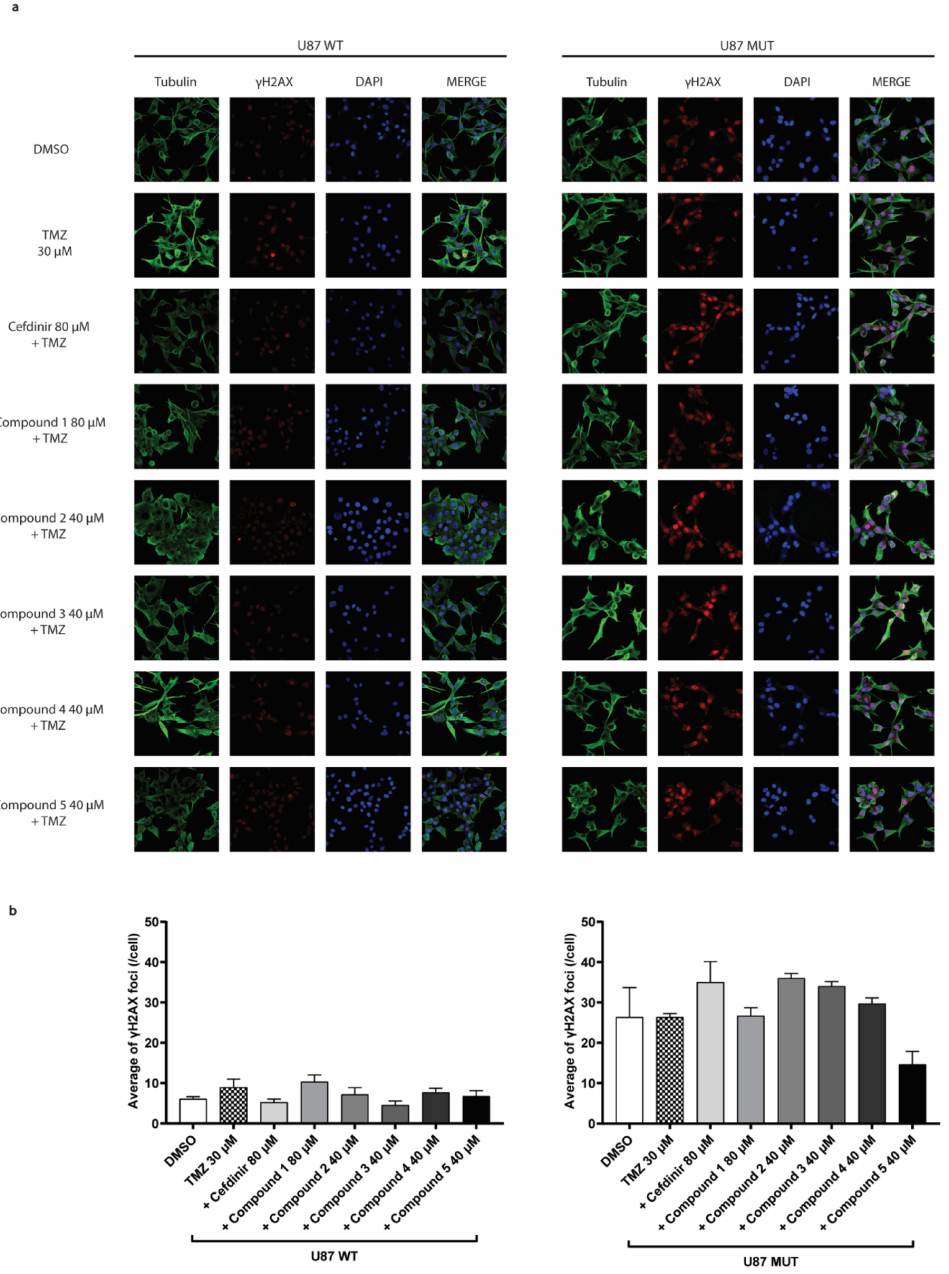
γH2AX immunofluorescence analysis in U87WT and MUT treated with RNaseH2 inhibitors and TMZ. (**a**) Immunofluorescence images of U87WT (left) and U87MUT (right) cells treated for 2 h with DMSO, 30 µM TMZ, and TMZ in combination with the highest concentration of RNaseH2 inhibitors. Cells were stained for tubulin (green), γH2AX (red), and DAPI (blue). (**b**) Quantification of γH2AX foci per cell. Bars represent the average number of foci per cell ± SD from at least four images. Statistical analysis was performed using one-way ANOVA. No statistically significant differences were observed.

To further investigate whether the observed synergy between RNaseH2 inhibitors and TMZ increased DNA damage, we performed γH2AX immunofluorescence staining following two hours of treatment with TMZ alone or in combination with the inhibitor molecules (Fig. 4a). In U87MUT cells, which are known to exhibit elevated basal levels of DNA damage^28^, we observed a trend toward an increase in γH2AX foci upon combination treatment compared to TMZ and DMSO (Fig. 4b). In the U87WT cells, the overall number of foci was lower, and the difference between the combination and the TMZ monotherapy was less pronounced (Fig. 4b). Notably, an increase in γH2AX foci was observed only with compound 1 in combination with TMZ. However, in both genotypes, the differences were not statistically significant.

### Combined treatment with TMZ and RNaseH2 inhibitor candidates is not toxic to non-cancer cells (human fibroblasts)

**Fig. 5:**
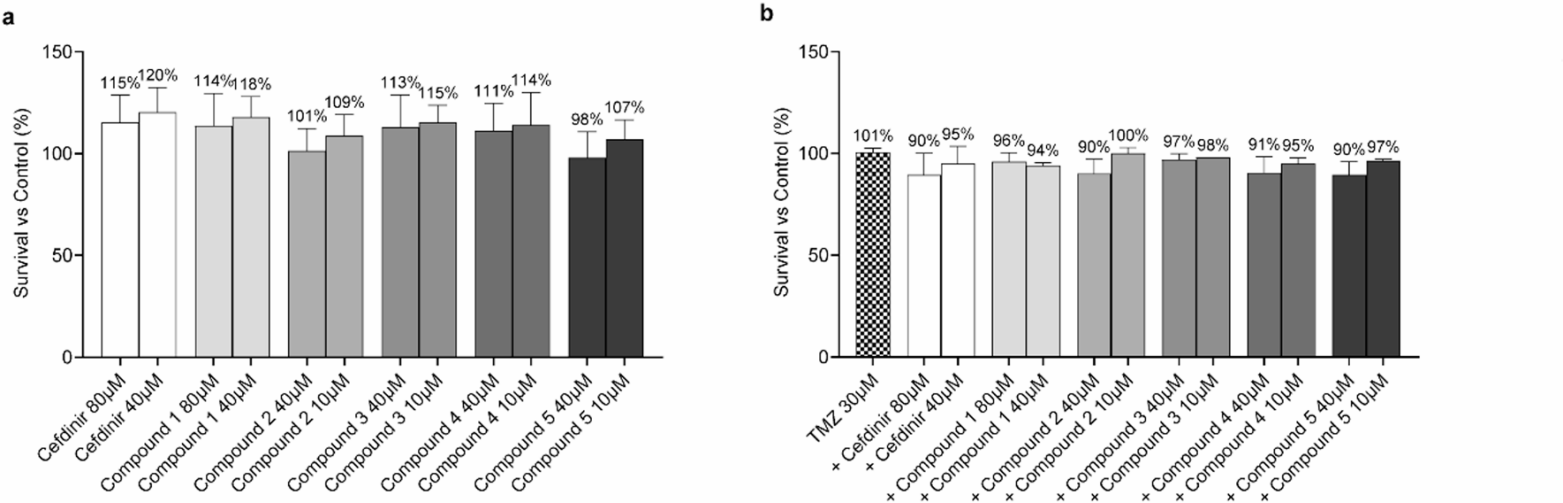
Toxicity assay on non-cancer human fibroblast cell line with the RNaseH2 novel inhibitor candidates. Cells were treated for 72 h with (**a**) Inhibitor treatment alone, and (**b**) in combination with 30 µM TMZ (+). Data are normalized to vehicle-treated control (DMSO), and mean survival percentages are given above the bars. Error bars indicate ± SD of at least three independent experiments. No statistically significant differences were observed between treatment groups (*p* > 0.05) using one-way ANOVA.

To investigate the potential toxicity of our compounds on non-cancerous cells, either as monotherapy or in combination with TMZ, we exposed human fibroblasts to the same inhibitor concentrations used for glioma cells. The viability results for fibroblasts treated with individual inhibitors are presented in Fig. 5a. At the tested concentrations, no significant toxicity was observed, indicating a favorable safety profile for the inhibitors. Figure 5b illustrates fibroblast viability following treatment with the inhibitors in combination with 30 µM TMZ. The results demonstrate minimal adverse effects on fibroblast viability, further supporting the specificity of these compounds for cancer cells. These findings suggest that the inhibitor candidates, whether used alone or in combination with TMZ, selectively target cancer cells without harming normal cells, highlighting their potential as safe therapeutic agents in GBM treatment.

### RNaseH2 inhibitor candidates display synergistic cytotoxicity with TMZ in patient-derived GBM organoid cells

**Fig. 6:**
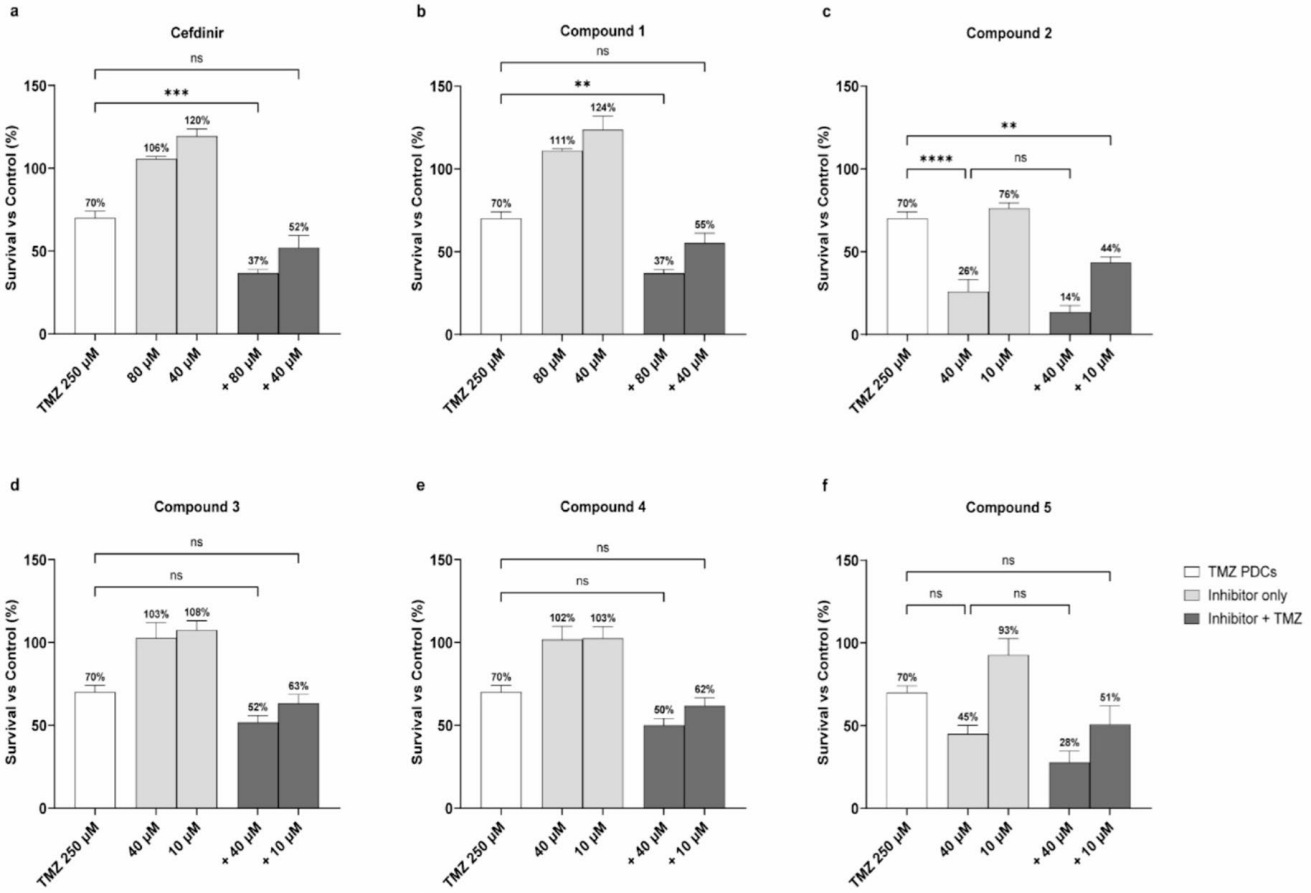
Single- and combinatorial treatments of TMZ and RNaseH2 inhibitor candidates in GBO-PDC. The cells were treated for 72 h with the RNaseH2 inhibitors at two concentrations alone and/or in combination with 250 µM TMZ. Plotted data are normalized to a vehicle-treated control (DMSO), and the survival percentage is depicted above the treatment bars as means ± SD of at least three independent experiments. Statistical significance has been calculated using one-way ANOVA followed by Tukey’s post hoc test (ns = non-significant; **p = 0.001–0.01; ***p = 0.0001–0.001; ****p < 0.0001).

After observing significant effects of RNaseH2 inhibitor candidates on glioma cell survival in combination with TMZ, we extended our investigation to GBO-PDC (IDH1 WT). Whereas U87WT and U87MUT are both MGMT negative and have high sensitivity to TMZ^29^, LC-MS/MS analysis confirmed robust MGMT expression in the GBO-PDC cells (data not shown). Consequently, higher TMZ doses were required to achieve comparable TMZ cytotoxicity to that observed in the U87MG cell lines (70% survival at 250 µM TMZ, Fig. 6). Single-agent treatment with compound 2 had a negative impact on the survival of the GBO-PDC cells (Fig. 6c). At 40 µM, this compound reduced GBO-PDC viability to 26% compared to vehicle-treated controls (Fig. 6c). Importantly, this concentration did not affect the viability of the survival of non-cancerous human fibroblasts (Fig. 5). Moreover, the cytotoxicity of compound 2 as a single agent was notably more pronounced in GBO-PDC cells than in U87MG cells (Fig. 3c). Notably, compound 2 exhibited the strongest effect in combination with TMZ (Fig. 6c), as both tested concentrations in cotreatment significantly reduced cell viability compared to single-agent treatments. Additionally, Cefdinir and compound 1 reduced the viability to 37% in combination with TMZ. We employed the same approach as for the U87MG cells to analyze drug synergy in GBO-PDC, and overall synergy scores are reported in Table 2.

**Table 2:** Synergy scoring between RNaseH2 inhibitor candidates and TMZ in GBO-PDC.

RNaseH2 Inhibitors	GBO-PDC	
	Chemotherapy	Synergy score
Cefdinir	+ TMZ 250 µM	16.95
Compound 1		20.03
Compound 2		−0.55
Compound 3		11.84
Compound 4		5.69
Compound 5		3.36

Synergistic combinations are marked in green (consensus synergistic value > 10; additive interaction − 10 to + 10; antagonism < −10).

### Enhanced synergy screening verifies results from the initial synergy analyses

**Fig. 7:**
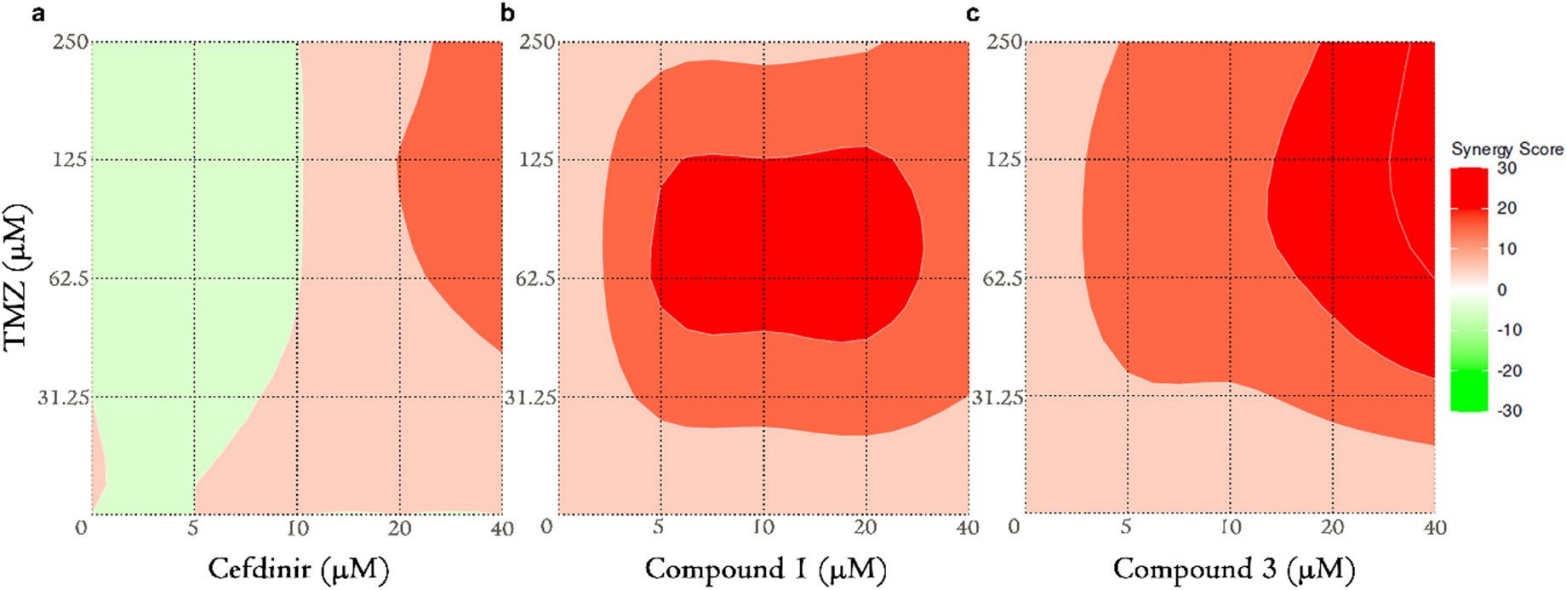
3D surface synergy plots after cotreatment of GBO-PDC cells with TMZ and cefdinir (**a**), compound 1 (**b**), and compound 3 (**c**). Heat bar- red/green scale- indicates synergistic/antagonistic drug interaction.

Based on the initial screening data, three inhibitor candidate-TMZ combinations resulted in synergistic action in the case of GBO-PDC (cefdinir, compound 1, and compound 3, Table 2). We further explored these cotreatments over a broader concentration range of both drugs (inhibitor candidates: 5, 10, 20 and 40 µM, TMZ: 31.25, 62.5, 125 and 250 µM) and generated more accurate synergy data visualized as 3D response surface plots (Fig. 7a-c). The initial indication of a synergistic effect between cefdinir and TMZ, observed at two inhibitor concentrations, was not fully supported upon further analysis, yielding an overall synergy score of 3.91 (Fig. 7a). This reduced effect may be attributed to reducing the maximum inhibitor concentration from 80 µM to 40 µM (Fig. 6). However, partial synergy data (Supplementary information table S1) revealed that the highest tested cefdinir concentration (40 µM) still exhibited synergy with TMZ at concentrations of 62.5 µM and above. In contrast, synergy was more strongly supported for inhibitor candidates compound 1 and compound 3 when combined with TMZ (Fig. 7b, c) with mean synergy scores of 15.54 and 18.94, respectively. These findings suggest that compound 1 and compound 3 are promising candidates for combinatory treatment with TMZ.

## Discussion

In this study, we identified several RNaseH2 inhibitor candidates with the potential to enhance current glioma therapies. RNaseH2 is a highly conserved enzyme complex comprised of three essential subunits and plays an important role in maintaining genomic integrity by degrading RNA-DNA hybrids and facilitating the removal of misincorporated ribonucleotides (rNMPs) during DNA replication. Failure to excise rNMPs can lead to genomic instability, a known driver of tumorigenesis. Additionally, inactivating mutations in genes encoding RNaseH2 subunits have been linked to Aicardi-Goutières syndrome (AGS), an inflammatory disorder associated with aberrant immune activation^30^. Elevated RNaseH2 expression has been reported in several cancers, including prostate and colorectal cancers, lung adenocarcinoma, and hepatocellular carcinoma^31–34^. Despite its multiple cancer-associated dysregulations, the therapeutic potential of RNaseH2 inhibition in gliomas remains largely unexplored.

Here, we provide evidence that RNaseH2 inhibitor candidates can reduce glioma cell survival, particularly in high-grade gliomas with IDH1 wild-type status, a subtype characterized by its aggressiveness and treatment resistance. Using both commercially available GBM cell lines (U87 IDH1 WT and MUT) and glioblastoma organoid-derived patient cell lines, we evaluated the efficacy of the inhibitor candidates alone and in combination with TMZ. Several candidates significantly reduced cell viability when combined with TMZ, especially in patient cells derived from glioblastoma organoids, underscoring their potential clinical relevance. Interestingly, combinatorial treatments with TMZ and either compound 1, compound 2 or compound 5 selectively decreased viability in the U87WT cell line, suggesting that IDH1 status influences drug response. Compounds 1 and 3 also exhibited a synergistic effect when combined with TMZ in IDH1 WT GBO-PDCs, highlighting their potential for further development in GBM therapy. Compound 2 was uniquely effective, significantly reducing cell viability at both tested concentrations in the U87WT cell line and in GBO-PDCs. The selective synergy observed in IDH1 WT backgrounds, particularly with the compound 2/TMZ combination, suggests that IDH1 mutation status modulates the efficacy of RNaseH2 inhibition, meriting further investigation in genetic models that further reflect glioma diversity. The discrepancy observed between the Presto Blue assay and the cell counting provides important insights into the nature of the cellular response to RNaseH2 inhibition. While the Presto Blue assay indicated substantial reductions in cell viability, the Trypan Blue–based cell counting showed only a moderate decrease in live cells under the same treatment conditions. This difference likely stems from the fact that metabolic activity, measured by Presto Blue, can decline during early stress responses or growth arrest, even in cells that retain membrane integrity and are still viable by Trypan Blue standards. To further investigate whether the observed synergy between RNaseH2 inhibitors and TMZ was associated with increased DNA damage, we examined treatment-associated γH2AX foci occurrence by immunofluorescence. Although a modest trend toward increased DNA damage was observed, particularly in U87MUT cells, these changes were not statistically significant. The lack of a robust γH2AX post-treatment signal, especially in U87WT cells, suggests that enhanced double-strand break formation may not be the dominant mechanism of synergistic glioma cell killing. These findings suggest that the observed synergy may not be exclusively due to enhanced DNA damage, but may involve alternative mechanisms, such as replication stress or cell cycle perturbation.

Cefdinir, an FDA-approved antibiotic used to treat conditions such as pneumonia, otitis media, and strep throat^35^, was demonstrated to significantly reduce the viability of GBO-PDCs with IDH1 wild-type status. Importantly, cefdinir is endowed with the ability to cross the blood-brain barrier (BBB), addressing a critical challenge in GBM treatment^36^. While the BBB permeability of the other RNaseH2 inhibitors identified in this study remains unknown, it highlights the need for further studies. Additionally, its enhancement of TMZ’s effects underscores its potential for drug repurposing, allowing for expedited preclinical and clinical trials^37^.

Beyond cytotoxicity, RNaseH2 inhibition may have important immunological consequences. This enzyme plays a crucial role in ribonucleotide excision repair, and its inhibition can lead to the accumulation of RNA-DNA hybrids. This, in turn, may lead to cytosolic DNA accumulation, which activates the cGAS-STING pathway^38^. Its activation induces type I interferon (IFN-I) signaling, which promotes dendritic cell activation, T cell priming, and recruitment of effector immune cells into the tumor microenvironment (TME)^14,39,40^. Additionally, RNaseH2 deficiency has been associated with increased secretion of immunomodulatory factors, such as CXCL10, IL-6, and TNF-α^16,20^, all of which may alter the TME by modifying immune infiltration, modulating myeloid cell populations, and reprogramming stromal components. These changes could render GBMs more immunogenic, improving responsiveness to immune-based therapies. Furthermore, the genomic instability associated with RNaseH2 inhibition may increase the tumor mutational burden (TMB), thereby enhancing tumor visibility to the immune system.

The genetic heterogeneity of GBMs, even within the same subtype, complicates the development of generalized treatments. Consequently, patient-derived models provide crucial insights into therapeutic efficacy and clinical translational potential. Our results suggest that IDH1 mutation status significantly impacts responsiveness to RNaseH2 inhibition, particularly in combination with TMZ, supporting a more personalized treatment approach to improve outcomes for glioma patients. In the future, these inhibitors/cotreatments will be tested in glioblastoma organoids, which offer a more accurate representation of human tumor heterogeneity at both inter- and intra-tumoral levels. Unlike animal models that rely on xenografts and may fail to capture the complexity of human tumors, patient-derived glioblastoma organoids preserve the original genetic, epigenetic, and microenvironmental characteristics of the patient’s tumor, making them a more physiologically relevant platform for research and drug testing^41^. In conclusion, this study highlights RNaseH2 inhibition as a promising strategy to improve outcomes in gliomas, particularly in IDH1 wild-type cases. Together, these results warrant further exploration of RNaseH2-targeted therapies within personalized glioma treatment strategies.

## Materials and methods

### Cell lines and cell culture

U87MG IDH1 WT (HTB-14) and IDH1 mutant (HTB-14IG) glioma cell lines and primary dermal normal human fibroblasts (PCS-201-012) were from ATCC and maintained in Dulbecco’s Modified Eagle Medium (DMEM, D6429) supplemented with 10% fetal bovine serum (FBS), 1% penicillin-streptomycin solution and 1% L-glutamine (Sigma-Aldrich). The GBO-PDC model; GBM, IDH-wildtype, CNS WHO grade 4, was established in Professor Magnar Bjørås’s laboratory at NTNU. GBO-PDC cells were maintained on plates pre-coated with Geltrex™ (Gibco, A1413201) in PDC medium: Knockout^®^ DMEM/F-12 (Gibco, 12660-012) supplemented with 1% penicillin/streptomycin (Sigma-Aldrich, P4333), 1×StemPro^®^ Neural Supplement (Gibco, A10508-01), 20 ng/ml recombinant human EGF (R&D Systems, 236-EG), 20 ng/ml recombinant human FGF2 (Peprotech, 100-18B) and 2 mM glutamine (Sigma-Aldrich, G7513). Upon passaging and cell seeding for viability assays, 10 µM ROCK inhibitor Y-27,632 dihydro-chloride (MCE, HY-10583) was added to the PDC medium for overnight incubation to prevent cell death.

### RNaseH2 enzyme purification

Recombinant RNaseH2 complexes were produced in E. coli BL21 (DE3) RIPL cells. Bacterial cultures were grown at 37 °C until OD600 = 0.6, followed by induction with 0.1 mM IPTG at 25 °C for two hours and further incubation at 16 °C for 14–16 h. Cells were harvested by centrifugation and lysed via sonication in PBS buffer supplemented with 0.2% Tween, 10 mM MgCl₂, complete protease inhibitor cocktail (Roche; 50 µL per gram of cell pellet), as well as 100 µg each of DNase I (NEB) and RNase A (Sigma-Aldrich). The cleared lysate was incubated with Glutathione-Sepharose 4B beads (GE Healthcare) for two hours at 16 °C to facilitate protein binding. Bound GST-tagged proteins^17^ and their interacting partners were eluted through cleavage with PreScission protease (GE Healthcare Life Sciences) at 4 °C for 14–16 h. The resulting protein complexes were further purified by size-exclusion chromatography (GE Healthcare Life Sciences) in a buffer containing 150 mM NaCl and 20 mM Tris-HCl (pH 7).

### RNaseH2 activity assays

RNaseH2 activity assay was performed at room temperature in 384-well plates (black OptiPlates, PerkinElmer). Briefly, the RNaseH2 enzyme was incubated with a UMP-containing DNA/RNA hybrid substrate. The substrate was prepared by annealing a 5’-Cy5-labelled DNA oligonucleotide containing a single UMP at the seventh position (5’-[Cy5]CTGGCA[rU]CACTGCGTCGACCTG-3’) to an excess of a complementary 3’ BHQ2-labelled oligonucleotide with a 2’-deoxyadenosine complementary to the ribouridine (5’-CAGGTCGACGCAGTGATGCCAG[BHQ-2]−3’). 30 nL of each compound (10 mM in DMSO, Table 3), was pre-dispensed into each well using a Labcyte Echo 550 acoustic dispenser. This was followed by addition of 10 µL purified, recombinant RNaseH2 (9 ng/mL) (Supplementary information figure S3) and 20 µL RNA/DNA substrate dissolved in assay buffer, using a BioTek Multiflo FX instrument. The assay buffer contained 20 mM Tris-HCl pH 8.0, 60 mM NaCl, 10 mM MgCl2, 0.005% NP40, and 1 mM DTT. The samples were incubated at room temperature for 20–30 min, and fluorescence was recorded using a Tecan SPARK plate reader at an excitation wavelength of 610^10^ nm and emission wavelength of 670^20^ nm.

**Table 3:** Overview of compound libraries tested in high-throughput screening assay.

Compound library	Number of compounds
Asinex PPI (Non-Macrocyclic)	966
BioMol Kinase Inhibitor Library	70
BioMol Known bioactives	79
ChemBridge Diversity	17255
ChemDiv ChemBioNet	16,308
Enamine Diversity	27698
Enzo Chemical Genomics	207
Enzo Pathway Targeting	205
Enzo Receptor De-Orphaning	70
MedChemExpress Epigenetics	2186
Prestwick Chemical Library (Approved Drugs)	1516
Prestwick Original Molecules (NOCE)	344
Selleck Bioactive Compound Library-I	989
Selleck Cambridge Cancer	352
Selleck Kinase	170
Selleck OncoSet	443
Selleck Tyrosine Kinase	171
Sigma LOPAC	1255
Tocris Mini	1110

### Inhibitors

RHI002 (N-(2-Furanylmethyl)−2-[(5,6,7,8-tetrahydro[1]benzothieno[2,3-d]pyrimidin-4-yl)thio]-acetamide), was obtained from AOBIOUS, INC (Gloucester, MA, USA). Cefdinir (1-[(3R,3aS,9aS)−3-Phenyl-decahydro-1 H-pyrrolo[3,2-b]azocin-1-yl]ethan-1-one diHCl) was from Active Scientific GmbH (Prien, Germany). Compound 1 (3-[2-(3-oxo-3,4-dihydro-2 H-1,4-benzothiazin-2-yl)acetamido]benzoic acid), compound 2 (5-[(1E)−2-(3,4-dihydroxyphenyl)ethenyl]benzene-1,3-diol), compound 3 (3-[2-(3-oxo-1,2,3,4-tetrahydroquinoxalin-2-yl)acetamido]benzoic acid), compound 4 (2,5-bis(2-phenylacetamido)benzene-1,4-dicarboxylic acid), and compound 5 (3-[[(5,5-dioxo-6 H-benzo[c][1,2]benzothiazin-9-yl)amino]methyl]−6-methoxy-1 H-quinolin-2-one), were purchased from AKOS Consulting & Solutions Deutschland GmbH (Lorrach, Germany). Temozolomide (TMZ) (T2577) was from Sigma-Aldrich Chemie GmbH (Darmstadt, Germany).

### Viability assay

A total of 3000 glioma cells or 5000 human fibroblasts per well were seeded in 96-well plates. In case of GBO-PDCs, 5000 cells were seeded/well in GeltrexTM precoated plates. After 24 h incubation at 37 °C, cells were treated with the inhibitor candidates (Cefdinir, compound 1–5), either alone or in combination with TMZ, for 72 h. To determine cell viability, a Presto Blue assay was conducted. The Presto Blue™ HS Cell Viability Reagent (Thermo Fischer, P50201) was prepared in culture medium according to the manufacturer’s instructions. After 30 min incubation at 37 °C (60 min for GBO-PDC), fluorescence intensity was measured using a BMG-Fluostar Omega plate reader (BMG Labtech). Each treatment was performed in triplicate and at least three independent experiments were performed for each cell line. The data were normalized to the vehicle-treated control (DMSO) and plotted using GraphPad Prism 10.

### Cell counting assay

A total of 5000 U87WT and U87 MUT cells per well were seeded in 96-well plates. After 24 h incubation at 37 °C, cells were treated with DMSO, TMZ (30 µM), Cefdinir (80 µM), Compound 1 (80 µM), Compound 2 (40 µM), Compound 3 (40 µM), Compound 4 (40 µM), and Compound 5 (40 µM), either alone or in combination with TMZ for 72 h. Cells were then collected, washed with PBS, and resuspended in 1 mL of DMEM. Live cells were stained with Trypan Blue (Invitrogen) and quantified by a Countess 3 automated cell counter (Invitrogen).

### Immunofluorescence analysis of γH2AX

A total of 5000 U87WT and U87MUT cells were seeded in a 96-well plate (µ-Plate 96 Well Round ibidi plate, Cat. No. 89601), and treated with DMSO, TMZ (30 µM) alone or in combination with Cefdinir (80 µM), Compound 1 (80 µM), Compound 2 (40 µM), Compound 3 (40 µM), Compound 4 (40 µM), and Compound 5 (40 µM). Following removal of the culture medium, cells were washed twice with PBS and subsequently fixed in 4% paraformaldehyde for 1 h at room temperature. After fixation, samples were washed once with PBS and permeabilized using 0.25% Triton X-100 in PBS. Cells were then blocked for 1 h at room temperature in blocking buffer containing PBS supplemented with 5% normal goat serum, 1% bovine serum albumin (BSA), and 0.1% Triton X-100. After blocking, samples were incubated overnight at 4 °C with primary antibodies diluted in a buffer composed of PBS with 1% normal goat serum, 1% BSA, and 0.1% Triton X-100. The next day, sections were washed three times with 1× PBS containing 0.1% Tween-20 (PBST), then incubated with appropriate fluorophore-conjugated secondary antibodies diluted in the same buffer used for primary antibody incubation (PBS supplemented with 1% normal goat serum, 1% BSA, and 0.1% Triton X-100) for 1 h at room temperature. After incubation, sections were washed three additional times with 1× PBST, mounted onto glass microscope slides, and allowed to air-dry overnight at room temperature. Nuclear counterstaining was performed using 4′,6-diamidino-2-phenylindole (DAPI; 1 µg/mL in PBS), followed by a brief wash in PBS. Slides were subsequently cover-slipped using ProLong™ Gold Antifade Mountant with DAPI (Thermo Fisher Scientific). The primary antibodies used were: anti-γH2A.X (phospho S139) (rabbit polyclonal, 1:500; Abcam, Cat. No. ab2893) and anti-βIII-tubulin (chicken polyclonal, 1:1000;Cat. No. ab9354, Merck). Corresponding secondary antibodies were obtained from Thermo Fisher Scientific: Alexa Fluor 488-conjugated goat anti-chicken IgY (1:1000; Cat. No. A-11039, RRID: AB_2534096) and Alexa Fluor 555-conjugated goat anti-rabbit IgG (1:1000; Cat. No. A-21428, RRID: AB_2535849). All fluorescent images were acquired using a confocal microscope (Airyscan Zeiss LSM880). For imaging the γH2AX marker, a Plan-Apochromat 40x/1.4 Oil DIC M27 objective (Carl Zeiss, Jena, Germany) was employed. Images were captured with a field size of 700 × 700 μm (2000 × 2000 pixels; pixel size: 0.35 μm) and a z-step interval of 0.5 μm. After three-dimensional rendering using the software Imaris 8.2 (Bitplane, Zurich, Switzerland), nuclei were identified by modelling spherical objects (‘spots’) with a diameter of 10 μm around all DAPI-positive areas. DAPI areas > 50% outside the imaging area were not considered for counting. The spots were used to define the intranuclear area by ‘masking’ the channel containing the γH2AX signal, setting the voxels outside the spots to a fluorescent signal equaling zero. Within the masked channel, high-intensity spots were identified by modelling spherical objects with a 0.8 μm diameter. Two random images with a relevant number of double-strand break spots were used as a reference measurement, in which spots were first identified using the automatic spot detection method provided by Imaris (‘quality’ filter, Gaussian filter with background subtraction), with an automatic thresholding algorithm. Next, the threshold defined in the two initial images was taken as a reference for ‘spots of interest’. All subsequent γH2AX spots were detected using the same threshold criterion after background subtraction to avoid any arbitrary judgement of what spot qualifies as relevant. The number of γH2AX spots was normalized to the number of DAPI nuclei in the image area.

### Statistical analysis and synergy scoring

Data are presented as means ± SD and were analyzed using one-way ANOVA followed by Tukey’s post hoc test to determine significant differences between treatments. All statistical analyses were performed using GraphPad Prism 10 software. p-values < 0.05 were considered statistically significant. For interactive analysis of drug interaction, the web server SynergyFinder + version 3 (www.synergyfinderplus.org) was implemented^42^. For consensus synergy scoring, the zero-interaction potency model (ZIP) was used. Z’-factors were calculated based on average and standard deviation values of samples containing DMSO solvent control (*n* = 16 per plate) and no-enzyme controls (*n* = 16 per plate)^43^.

## Supplementary Information

Below is the link to the electronic supplementary material.

Supplementary Material 1

## Data Availability

Numerical data from the robotic library screening can be obtained by contacting Dr. Torkild Visnes. torkild.visnes@sintef.no.
