# Supplementary material for: RNaseH2 inhibition potentiates temozolomide response in patient derived glioblastoma cells
Source: Sci Rep. 2025 Nov 24;15:41486. doi: 10.1038/s41598-025-25298-5 (PMC12644484; doi:10.1038/s41598-025-25298-5)
Supplement: Supplementary file 1 — Supplementary Material 1 [file 41598_2025_25298_MOESM1_ESM.docx]

*Supplementary information*

**RNaseH2 inhibition potentiates temozolomide response in patient derived glioblastoma cells**

Miroslava Kissova^1^, Judit Martinez Segarra^1§^, Tobias Solli Iveland^1,11§^, Marthe Vestvik^1^, Erlend Ravlo^1^, Wei Wang^1^, Lars Hagen^7^, Nina-Beate Liabakk^1^, Maria Camara-Quilez^1^, Miquel Arano Barenys^1^, Ole Solheim^5,6^, Bård Helge Hoff^8^, Eirik Sundby^9^, Magnar Bjørås^1,4,10^, Geir Slupphaug^1,2,7^ Torkild Visnes^3*^ and Alessandro Brambilla^1,2,7*^

1 Department of Clinical and Molecular Medicine, Norwegian University of Science and Technology NTNU, 7491, Trondheim, Norway

2 Clinic of Laboratory Medicine, St. Olavs hospital, 7491, Trondheim, Norway

3 Department of Biotechnology and Nanomedicine, SINTEF Industry, Trondheim, Norway.

4 Department of Microbiology, Oslo University Hospital and University of Oslo, Oslo, Norway

5 Department of Neuromedicine and Movement Science, NTNU, Trondheim, Norway

6 Department of Neurosurgery, St. Olav's University Hospital, Trondheim, Norway

7 PROMEC Core Facility for Proteomics and Modomics, Norwegian University of Science and Technology, NTNU, and the Central Norway Regional Health Authority Norway, 7491, Trondheim, Norway

8 Department of Chemistry, Faculty of Natural Sciences, Norwegian University of Science and Technology NTNU, Trondheim, Norway

9 Department of Material Science, Norwegian University of Science and Technology, NTNU, 7491, Trondheim, Norway

10 Center of Embryology (CRESCO), University of Oslo, 0313, Oslo, Norway

11 Department of Oncology, Akershus University Hospital, 1478, Lørenskog, Norway

§ Contributed equally

Corresponding Author

* Alessandro Brambilla Department of Clinical and Molecular Medicine, Norwegian University of Science and Technology NTNU, N-7491, Trondheim, Norway. E-mail: Alessandro.brambilla@ntnu.no

*Torkild Visnes Department of Biotechnology and Nanomedicine, SINTEF Industry, Trondheim, Norway. E-mail: torkild.visnes@sintef.no

1. **Buffer optimization**


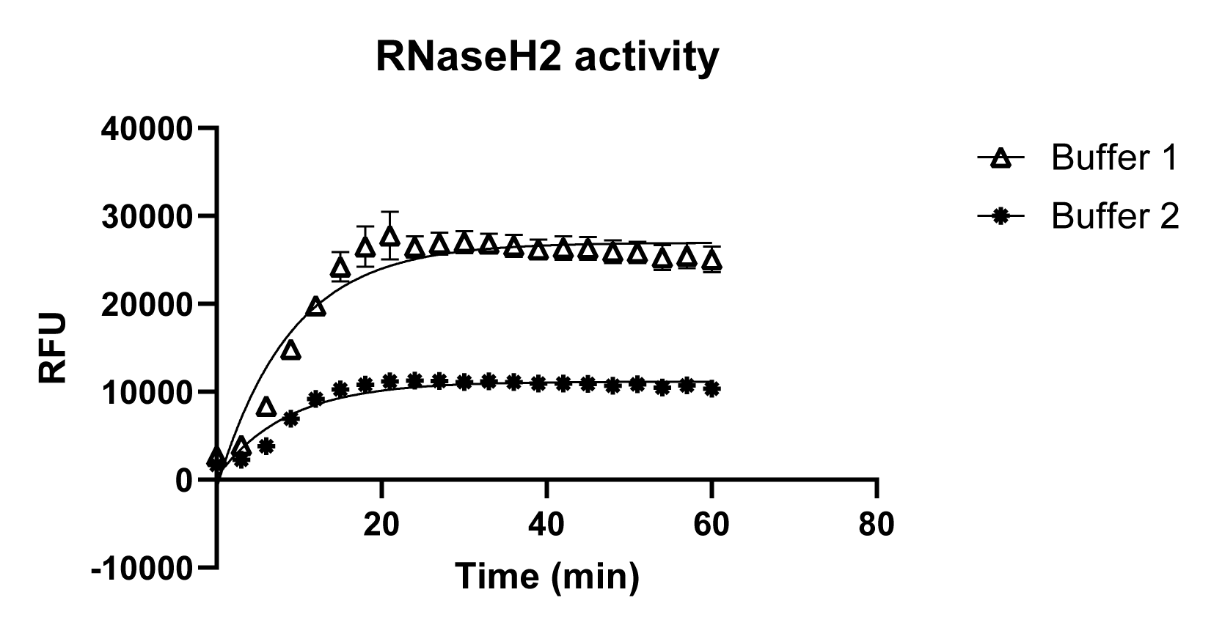


Figure S1. RNaseH2 activity was assessed using two different reaction buffers. Buffer 1 contained 20 mM Tris-HCl (pH 8.0), 60 mM NaCl, 10 mM MgCl₂, 0.005% NP-40, and 1 mM DTT. Buffer 2 contained 50 mM Tris-HCl (pH 8.0), 75 mM NaCl, 3 mM MgCl₂, 0.0025% Tween-20, and 1 mM DTT. The assay was performed with 50 ng/ml of purified RNase H2 enzyme. RNaseH2 showed higher activity in buffer 1 and was used for the high-throughput screening.

1. **Cell counting assay**

Figure S2. Cell counting assay: U87WT (a) and MUT (b) were treated for 72 h with DMSO, 30 µM TMZ, inhibitors alone, or in combination with TMZ. Following Trypan Blue staining, live cells were quantified using Invitrogen Countess 3 Automated Cell Counter.

1. **RNaseH2 purification**

**
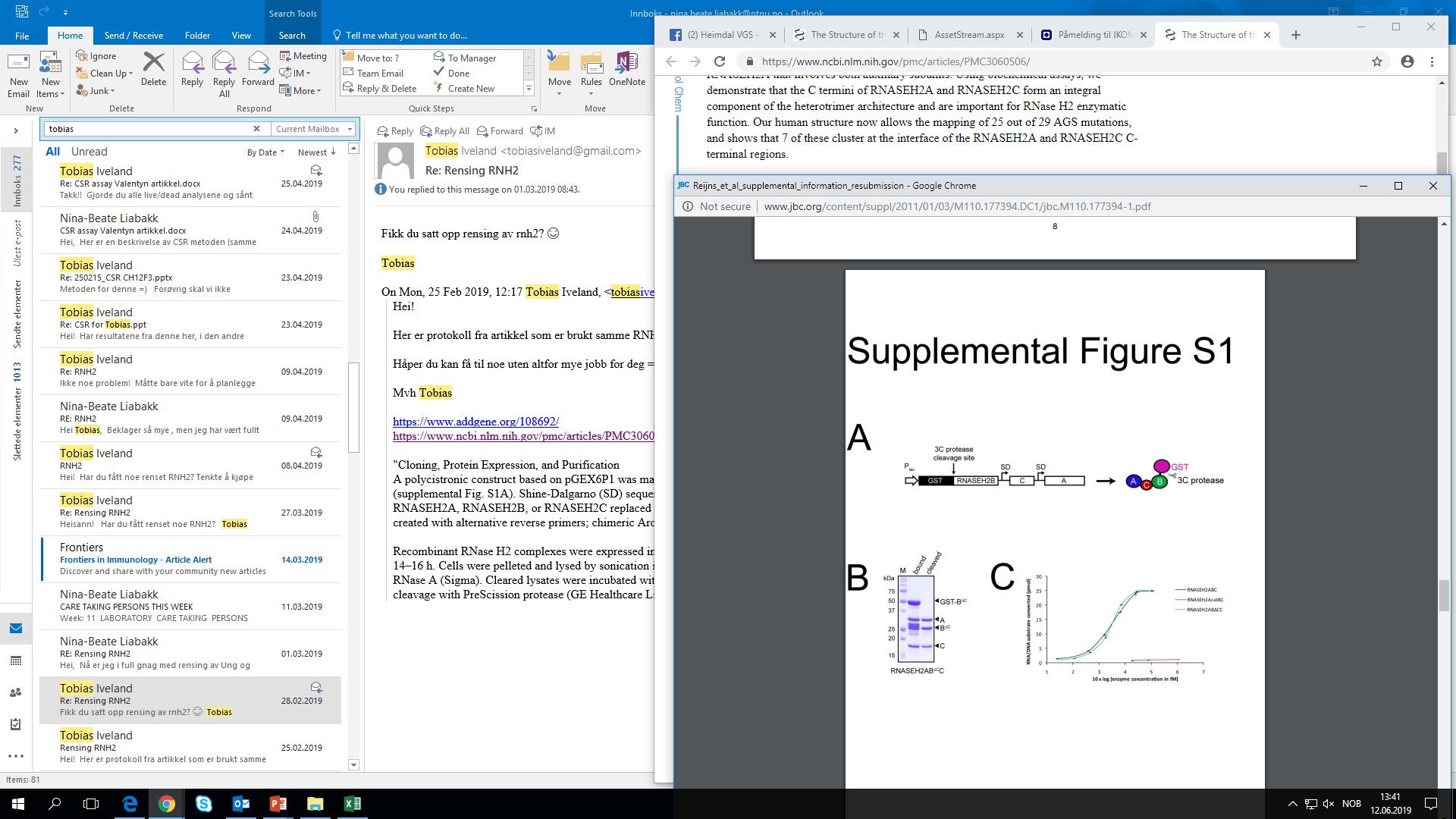
**

a

**
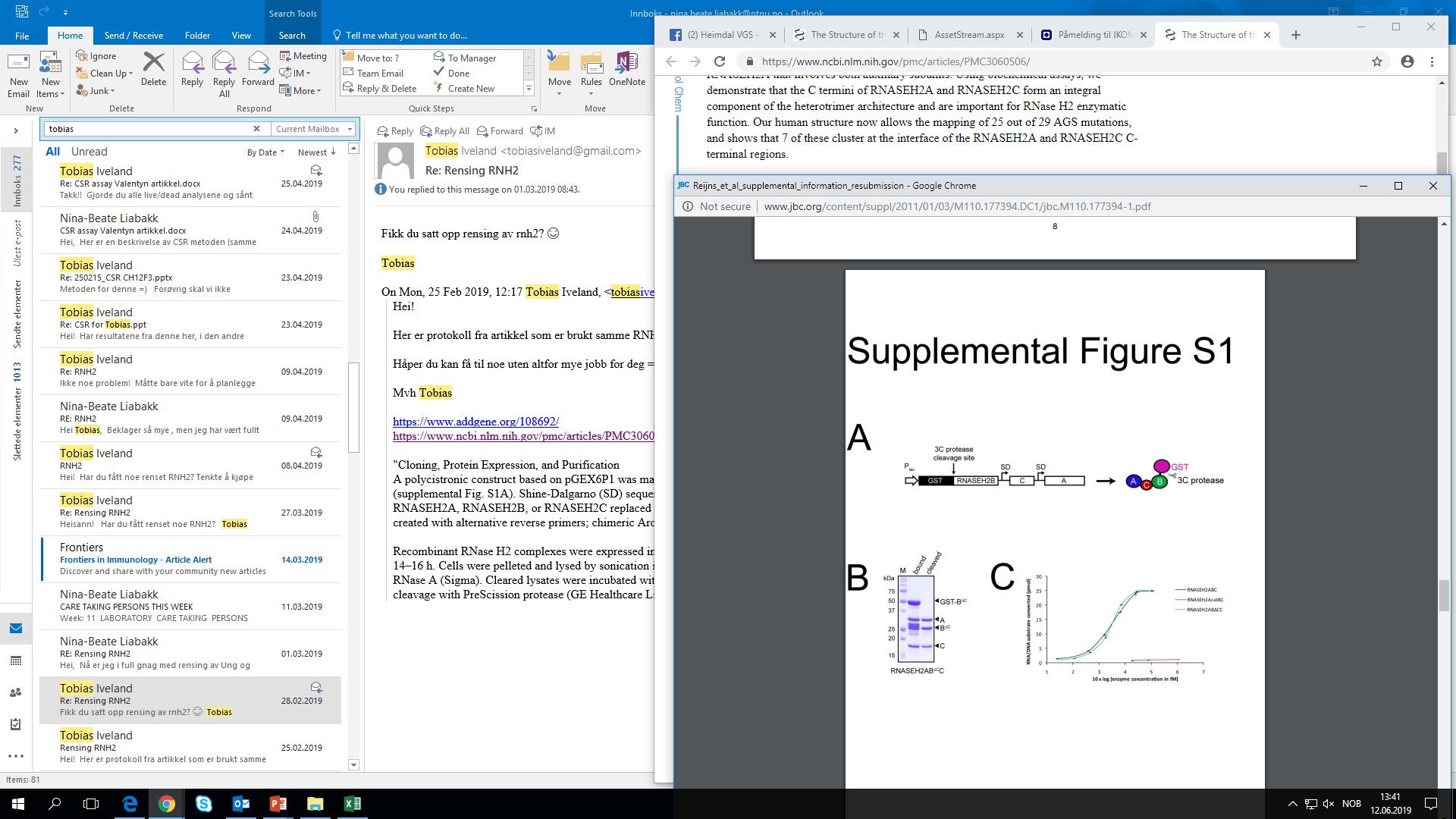
**

b

**S FT W1 W2 E1 E2 E3 E4**


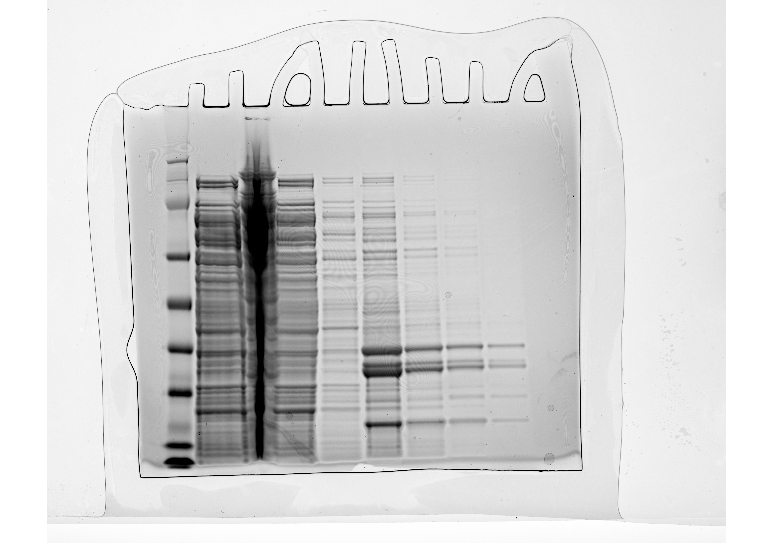


**S FT W1 W2 E1 E2 E3 E4**

**97**

**64**

**51**

**39**

**28**

**19**

**14**


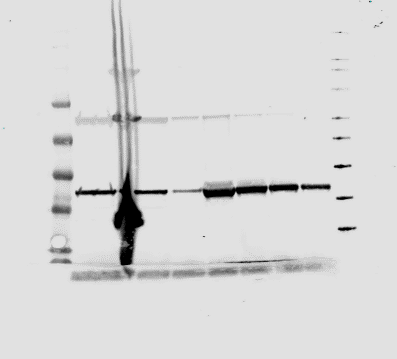


c

**Size-exclusion chromatography, fraction E2**

**Size-exclusion chromatography, fraction E1**


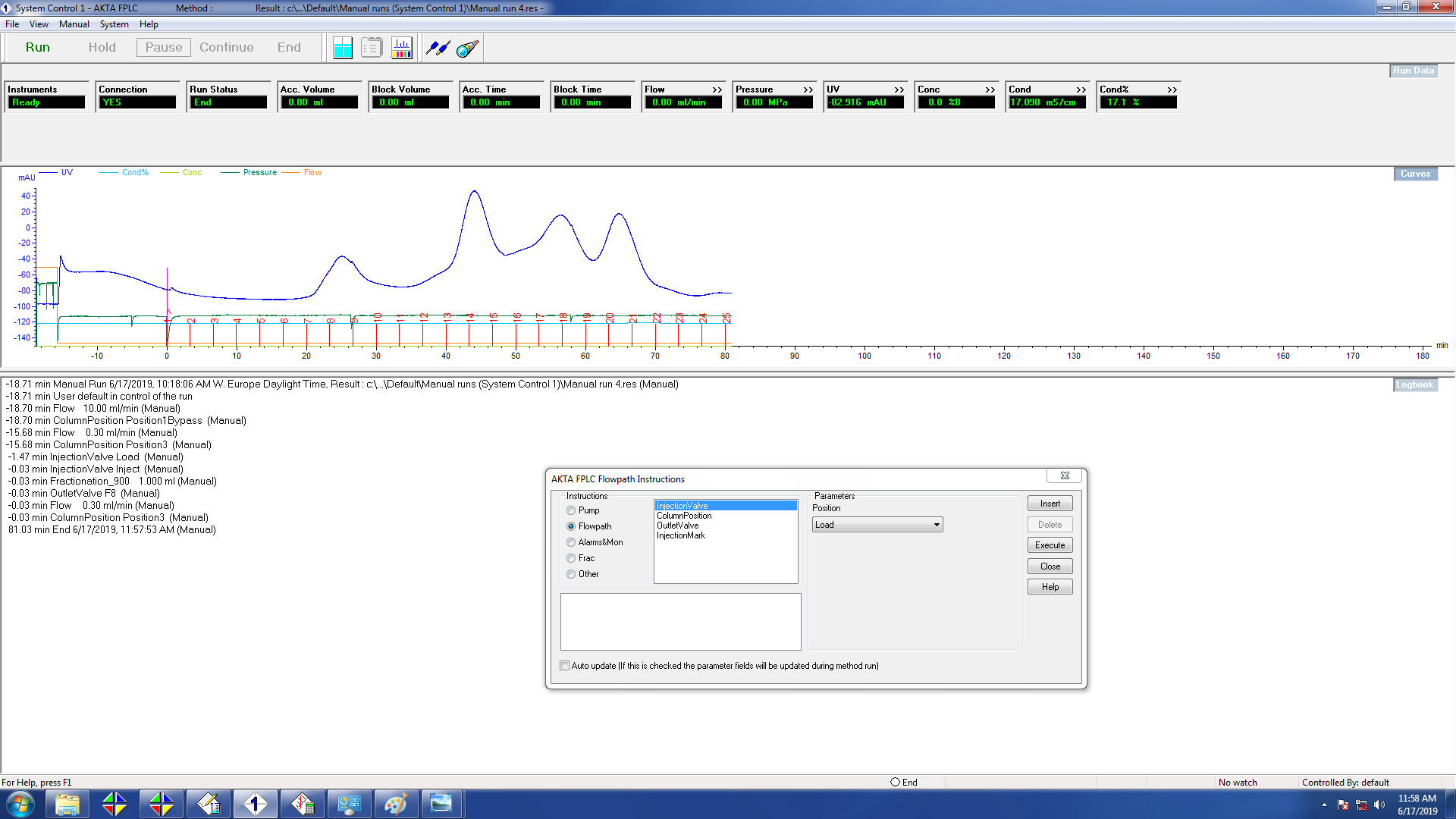

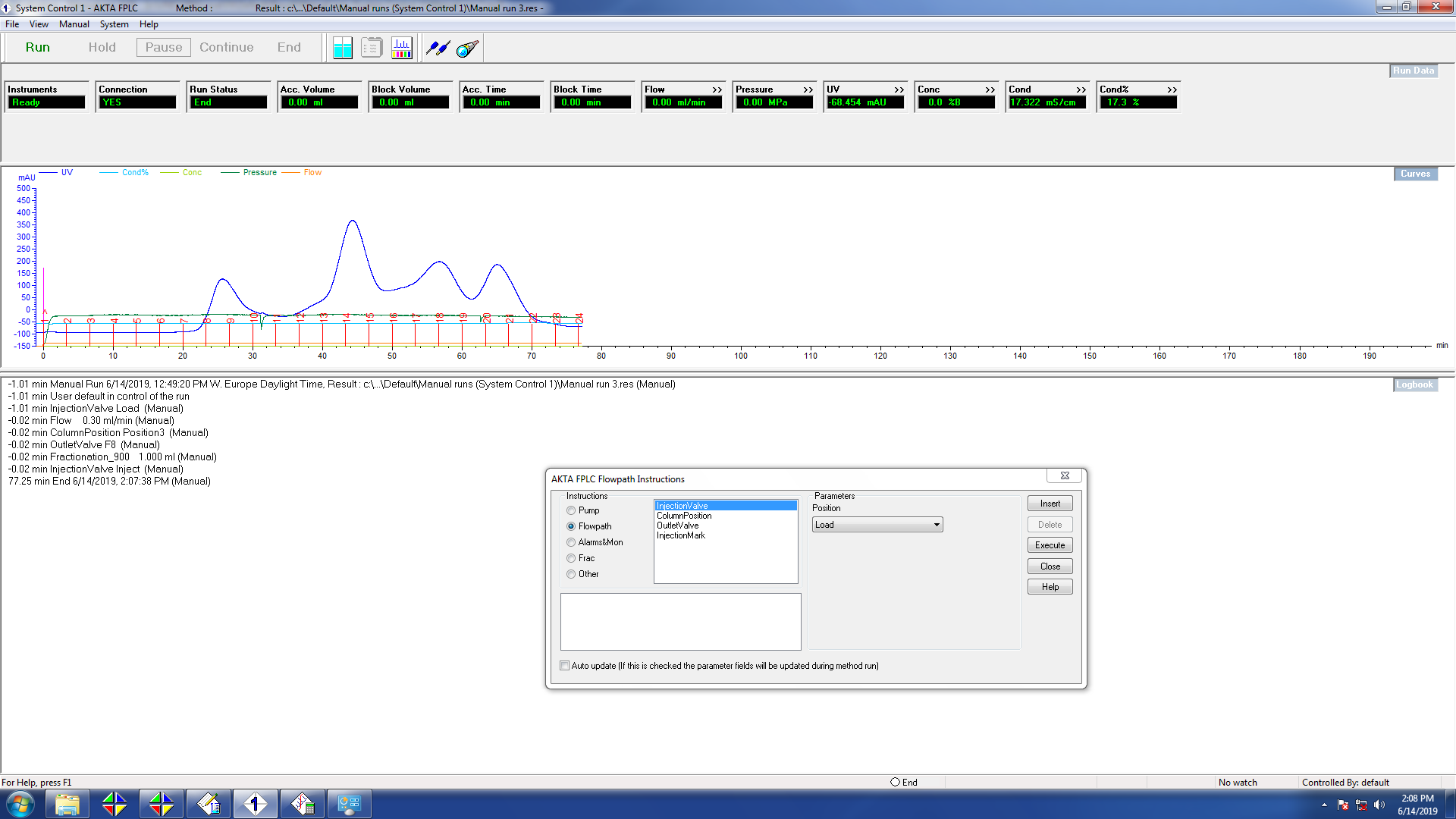


RNaseH2 trimer

RNaseH2 trimer


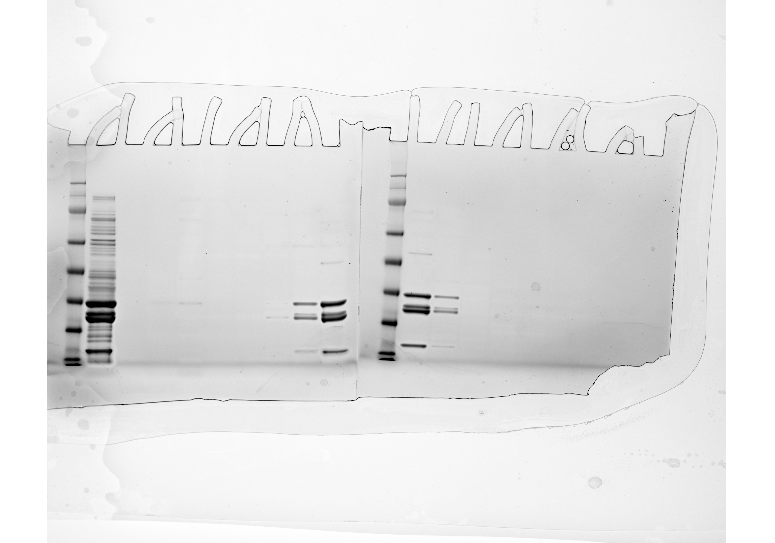


**E1 7 8 9 10 11 12 13 14 15 16**

**97**

**64**

**51**

**19**

**39**

**28**


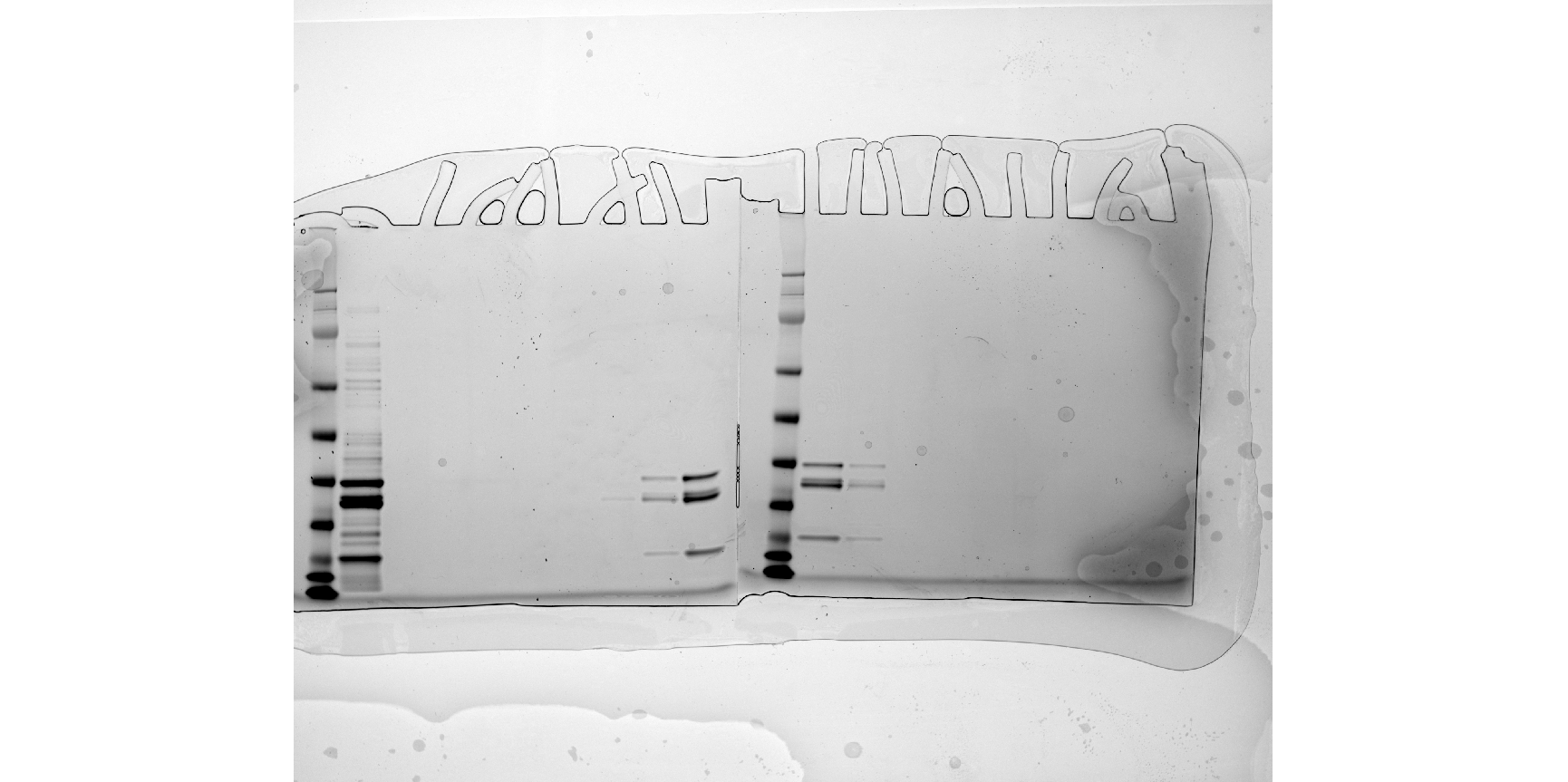


**E2 7 8 9 10 11 12 13 14 15 16**

d

**
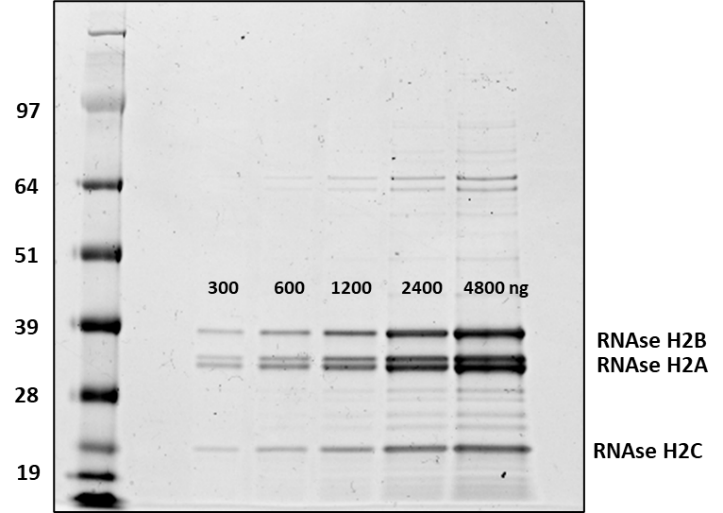
**

Figure S3. RNAseH2 purification: a) A polycistronic construct based on pGEX6P1 was made to express the human RNaseH2 trimer, allowing simultaneous expression of GST-tagged RNASEH2B, and untagged RNASEH2A and RNASEH2C. b) Glutathione-Sepharose 4B (GE17-0756-01) affinity chromatography Blue Gel and Western Blot of the purified RNaseH2 complex. c) Purification of E1 and E2 fractions by size-exclusion chromatography after PreScission protease incubation to remove the GST tag. d) All batches with purified RNaseH2 have been pooled together and checked by Mass Spectrometry.

**Table S1. Synergy scoring table**

| **Cefdinir [μM]** | **TMZ [μM]** | **ZIP_fit** | **ZIP_ref** | **ZIP_synergy** |
| --- | --- | --- | --- | --- |
| 0 | 0 | 0 | 0 | 0 |
| 5 | 0 | -3 | -3 | 0 |
| 10 | 0 | -2 | -2 | 0 |
| 20 | 0 | -1 | -1 | 0 |
| 40 | 0 | 1 | 1 | 0 |
| 0 | 31.25 | 10 | 10 | 0 |
| 5 | 31.25 | 3.569231 | 4.464864 | -0.895633002 |
| 10 | 31.25 | 5.46559 | 4.464864 | 1.000726532 |
| 20 | 31.25 | 7.008728 | 4.464864 | 2.543864579 |
| 40 | 31.25 | 12.25135 | 4.464889 | 7.786460398 |
| 0 | 62.5 | 8 | 8 | 0 |
| 5 | 62.5 | 3.023663 | 8.796747 | -5.773083797 |
| 10 | 62.5 | 8.483921 | 8.796747 | -0.312826123 |
| 20 | 62.5 | 17.24986 | 8.796747 | 8.453116368 |
| 40 | 62.5 | 22.05649 | 8.796771 | 13.25972201 |
| 0 | 125 | 15 | 15 | 0 |
| 5 | 125 | 8.613094 | 16.43105 | -7.817951838 |
| 10 | 125 | 15.82788 | 16.43105 | -0.603163316 |
| 20 | 125 | 26.7887 | 16.43105 | 10.35765823 |
| 40 | 125 | 30.68222 | 16.43107 | 14.25115544 |
| 0 | 250 | 31 | 31 | 0 |
| 5 | 250 | 26.99564 | 29.21104 | -2.21539663 |
| 10 | 250 | 29.16365 | 29.21104 | -0.047387077 |
| 20 | 250 | 36.17086 | 29.21104 | 6.959821178 |
| 40 | 250 | 44.81273 | 29.21106 | 15.60167447 |

Table S1. Synergy scoring of Cefdinir and TMZ. The highest Cefdinir inhibitor concentration results in synergy with TMZ starting from 62.5 μM. In green, the synergistic drug combinations (ZIP synergy score) are highlighted.
